# Femoral venous oxygen saturation is no surrogate for central venous oxygen saturation

**DOI:** 10.1186/cc9460

**Published:** 2011-03-11

**Authors:** A Van der Schors, P Van Beest, H Liefers, L Coenen, R Braam, P Spronk

**Affiliations:** 1 Gelre Hospitals, Apeldoorn, the Netherlands; 2 University Medical Center, Groningen, the Netherlands

## Introduction

Shock is defined as global tissue hypoxia secondary to an imbalance between systemic oxygen delivery (DO_2_) and oxygen demand (VO_2_), reflected by mixed venous oxygen saturation (SvO_2_). Intervention based on markers of tissue hypoperfusion may improve outcome. Central venous oxygen saturation (ScvO_2_) has been used as a surrogate marker for SvO_2_. In order to monitor ScvO_2 _during resuscitation, an internal jugular or subclavian line must be inserted. However, sometimes the femoral vein is the preferred or only possible site for access. The purpose of our study is to determine whether ScvO_2 _and femoral venous oxygen saturation (SfvO_2_) can be used interchangeably.

## Methods

A single-center, prospective, controlled, observational study was conducted at the Gelre Hospitals Apeldoorn. One hundred stable cardiac patients who underwent elective right heart catherization in daycare served as a control group. In the study group (high-risk surgery, ASA >2, *n *= 30) we determined SfvO_2 _and ScvO_2 _simultaneously at the start (T = 0) and at the end (T = 1) of the procedure. For each time point we calculated the agreement and difference between both values.

## Results

Control group: ScvO_2 _and SfvO_2 _correlated significantly (*r *= 0.67, 95% CI = 0.50 to 0.80; *P *< 0.0001) with large limits of agreement (bias 2.0 ± 7.1; -11.8 to 15.9). In the surgical patients at T = 0, mean values were similar (SfvO_2 _82.5 ± 6.6% vs. ScvO_2 _81.1 ± 8.1; *P *= 0.28). According to Bland-Altman analysis, the mean bias between ScvO_2 _and SfvO_2 _was 2.7 ± 7.9% and 95% limits of agreement were large (-12.9% to 18.2%), while correlation between ScvO_2 _and SfvO_2 _was significant (*r*^2 ^= 0.35; *P *< 0.01). At both time points SfvO_2 _and ScvO_2 _did not correlate significantly (*P *= 0.26 and *P *= 0.66 respectively) with similar negligible *r*^2^. Univariate analysis did not show any parameter (including dosages of dopamine or norepinephrine, total infusion, fluid balance, FiO_2_, type of surgery, lactate, and haemoglobin level) affecting either SfvO_2 _or ScvO_2_. Results were similar for changes in SfvO_2 _and changes in ScvO_2_.

## Conclusions

Absolute values of SfvO_2 _are unsuitable as surrogate for absolute values of ScvO_2_. Also, the trends of both values are not interchangeable. Further studies should investigate the effects of treatment on SfvO_2_.

